# Impact of HIV Co-Infection on Clinical Presentation in Patients with TB and Correlation of the Findings with Level of Immune Suppression

**Published:** 2018-03

**Authors:** Rajendra Prasad Takhar, Kiran Mirdha, Gopal Purohit, Lokesh Maan, Mahendra Kumar Bainara

**Affiliations:** 1 Department of Respiratory Medicine, Govt Medical College, Kota (Raj.), India; 2 Department of Gynaecology and Obstetrics, Dr SN Medical College, Jodhpur (Raj.), India; 3 Department of Respiratory Medicine, Dr SN Medical College, Jodhpur (Raj.), India; 4 Department of Respiratory Medicine, Mahatma Gandhi Medical College, Kota (Raj.), India; 5 Department of Respiratory Medicine, R N T Medical College, Udaipur (Raj.), India.

**Keywords:** HIV infection, TB, Clinical presentation, Immune-suppression, Acid Fast Bacilli

## Abstract

**Background:**

The Human Immunodeficiency Virus (HIV) has long been known to alter the clinical presentation of tuberculosis (TB), which varies according to the time of occurrence of TB infection and the level of immunodeficiency. Identifying variations in clinical features in HIV-TB coinfection might be helpful in settings with limited diagnostic facilities. The aim of this study was to assess the clinical presentation of TB in HIV coinfection and associate clinical findings with level of immune suppression (CD4 count).

**Materials and Methods:**

In this prospective, cross-sectional observational study 110 patients having TB-HIV co-infection were assessed for clinical presentation and correlation with CD4 count. The study setting was a tertiary care teaching hospital. Patients were categorized in three group based on CD4 counts as group I: ≤ 100 cells/cmm, group II: 101–200 cells/cmm and group III: > 200 cells/cmm.

**Results:**

110 patients were enrolled, 70% had CD4 cell count < 200 cells/mm^3^. Mean age and CD4 cell were 33.82±8.79 years and 181.7cells/cmm, respectively. Most common form of tubercular involvement was pulmonary (56.4%) followed by combined pulmonary and extra-pulmonary involvement (28.2%) and exclusively extra-pulmonary (15.5%). No significant intergroup difference was observed in site of involvement among three groups (p=0.700). Cough (91.8%) followed by low grade fever (85.5%), anorexia (82.7%) and weight loss (66.4%) were the commonest presenting symptom without any significant inter group difference. 70.9% patients were in undernourished category and 53.6% were febrile on examination. Sputum negative TB was noted 53.8%. 72.0% of patients with CD4 counts ≤100 had sputum negative TB as compared to sputum positive TB (28%).

**Conclusion:**

Due to varied clinical presentation of TB in HIV patients, ample knowledge of the clinical spectrum at different levels of immunosuppression is absolutely necessary to identify such patients early.

## INTRODUCTION

Tuberculosis (TB) is the leading cause of death from any single pathogen worldwide. This situation has worsened following advent of the Human Immune deficiency Virus (HIV) pandemic, leading to a dramatic increase in the number of TB cases globally (1). HIV and TB have long been known to have a close and complex association, with each having the potential to alter the natural course of the other. The term ‘co-epidemic’ or ‘dual epidemic’s is often used to describe their relationship. Also referred as “cursed duet” or “deadly duo”, this dual epidemic is becoming a continuously increasing global emergency especially in the developing and underdeveloped countries (2). There exists a harmful bidirectional and synergistic interaction (syndemic) between both these diseases, speeding the progression of illness which has added dramatically to the suffering and death caused by each disease alone (3). Because of this deadly syndemic of one viral and one bacterial, one recently emergent and one ancient—almost 4 million persons die every year, most of whom live in developing nations (4, 5).

The impact of HIV on the TB pandemic has been extensive. World Health Organization (WHO) estimates that in 2014, worldwide 9.6 million people have fallen ill with TB and an estimated 1.2 million people living with HIV developed TB. In 2014, TB killed some 1.5 million people of which 0.4 million were HIV-positive. One in three HIV deaths was due to TB (6). India has the highest total burden of TB in the world with an estimated 2.2 million incident cases in 2014 including 0.11 million cases of HIV-TB co-infection (7).

HIV-infection is the strongest risk factor for progression of latent TB infection to active disease (4). About a third of the human population is estimated to be latently infected with *Mycobacterium TB* (*M. tuberculosis)* but the lifetime risk of active TBTB is only 5–10% in persons with TB infection alone, whereas the annual risk of active TB is 5 to 15% in persons co-infected with HIV and TB. It is estimated that about 60–70% of HIV positive persons will develop TB in their lifetime (1). Patients with co-infection also have a higher case fatality rate and lifetime risk is above 30%. HIV is one of the most important factors contributing to the increased incidence of TB globally (4) due to either primary infection (8), re-infection (4, 9), increasing the risk of progression from one stage of TB to the next, or progression from latent infection (clinically quiescent) to clinical disease (reactivation) (10), as control of TB in an individual depends on an intact cellular immune response (11). TB itself can also accelerate the course of HIV infection by lowering the CD4 cell count (12) and increased HIV replication at sites of disease affected by TB such as lung parenchyma and pleural fluid (13).

Immune control of *M. tuberculosis* depends on both innate and adaptive cell-mediated immunity (14). Untreated HIV infection has been known to weaken the immune system either by severely depleting the CD4 cells (15) or by CD4 T-cell dysfunction as evidenced by reduced IL-2 or IFN-c production (16), and this in turn increases susceptibility to infections, including TB. Thus, the incidence of TB in HIV-infected patients is closely correlated to the severity of immunosuppression (17).

TB can occur in a person with HIV infection at any level of immunosuppression, though the clinical manifestations of TB are variable with the level of immune suppression, the time of, occurrence and diagnosis of either disease. Patients with TB-HIV co-infection and relatively intact immune system are more likely to have typical features of TB, while severely immune-suppressed patients are more likely to have atypical and disseminated forms of TB (2, 4, 18), and more prevalent extrapulmonary TB that too with concurrent pulmonary disease (3, 19).

The diagnosis of TB is notoriously challenging among HIV infected persons due to this atypical clinical presentation, substantially impaired diagnostic accuracy of sputum smear microscopy (more likely to have negative sputum AFB smears) and chest radiography. Further nonavailability of culture proven facilities, Polymerase Chain Reaction (PCR) test, and other newer diagnostic methods in resource-limited settings makes it difficult to diagnose such cases (5, 20–22).

Though, few studies have been conducted in India describing the clinical features of pulmonary TB in HIV seropositive patients (5, 23), these studies have simply compared the clinical and Chest X-Ray (CXR) findings of TB among HIV-infected and non-HIV-infected patients. Despite, a series of questions about the impact of HIV on the clinical presentation, remain unanswered. We planned this study to understand variations in clinical presentation of TB with different levels of immunosuppression in patients with HIV-TB co-infection.

## MATERIALS AND METHODS

This analytical cross-sectional study was conducted in TB and Chest hospital, Bari (Udaipur), Rajasthan, which is tertiary level care centre for patients with TB. Patient's ≥15 years of age with current diagnosis of TB and HIV positive status, who were willing to participate in to the study were enrolled over a period of 18 months. Patients receiving long term steroids, or other immunosuppressive therapy, diabetes mellitus, primary immune deficiencies and other causes of immune-suppression, were excluded from the study. After signing informed consent form, each participant completed a face to face structured questionnaire concerning socio-demographic characteristics, and clinical and laboratory data were collected into a structured datasheet.

Diagnosis of current TB was based on clinical characteristics, history of any contact with TB patients and supportive investigations either alone or in combination (including sputum smear/, culture for acid fast bacilli (AFB), Chest X-ray, Tuberculin/Mantoux test, relevant fluid (pleural fluid, CSF etc.) analysis and tissue biopsy / cytopathological examination of relevant site).

Absolute CD4+ counts were obtained using a B D FACS System. Patients were categorized in to three groups based on CD4 cell counts as group I (CD count ≤ 100 cells/mm^3^), group II (CD4 count 101–200 cells/mm^3^) and group III (CD count > 200 cells/mm^3^).

Routine blood and urine examinations were carried out in all the participants. Other relevant investigations including ultrasonography of abdomen and chest, Contrast-Enhanced Computed Tomography (CECT) of abdomen and chest were performed when required in particular set of patients.

### Ethical Statement

Ethical clearance for the study was obtained from the Institutional ethical committee approved the study protocol. Written informed consent obtained from all the patients before participation in to the study.

### Statistical Analysis

Continuous data was presented as mean (±SD) and categorical data was presented as frequency and percentages. One-way ANOVA was used for intergroup comparison of mean of continuous variables in three groups. Chi square was used for significance determination in categorical variables. P value < 0.05 was considered significant. Data analysis was performed with Microsoft excel 2016 and SPSS statistical software version 15 for analysis.

## RESULTS

**Figure 1.**
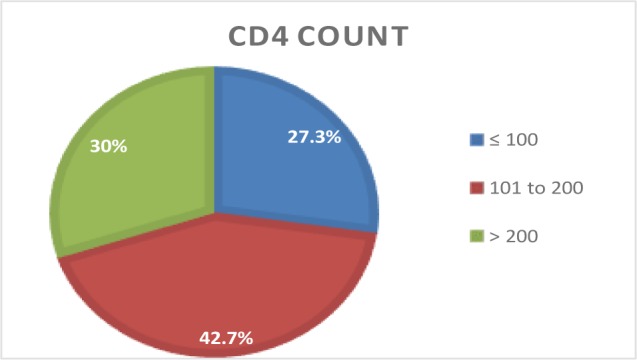
Percentage count of three groups of CD4 count.

In total 110 patients with co-infection of HIV and TB, most patients (42.7%) had CD4 counts between 101 to 200 (Figure 1). Compared to females, males were affected more frequently with significant difference observed amongst groups (p=0.013), while the duration of combined illness did not vary significantly among the groups (Table 1). The demographic and essential medical history characteristics (Table 2) in three groups shows that mean age of the patients was not significantly different in three groups based on CD4 count.

**Table 1.** Distribution of CD4 counts and duration of illness in two genders

**Parameter**	**Gender**		**P value**
	**Male (%)**	**Female (%)**	
**CD4 counts**			
**<100**	15 (19.2)	15 (46.9)	0.013^*^
**101–200**	37 (47.4)	10 (31.3)	
**>200**	26 (33.3)	7 (21.9)	
**Duration of Illness**			
**≤ 3 months**	28 (35.9)	12 (37.5)	0.984
**4 – 12 months**	37 (47.4)	15 (46.9)	
**> 12 months**	13 (16.7)	5 (15.6)	

**Table 2.** Demographic and medical history characteristics in three groups of CD4 cell count

**Characteristic**	**Total (n=110)**	**CD4≤100 (n=30)**	**CD4101–200 (n=47)**	**CD4>200 (n=33)**	**P value**
**Mean age (years)**	33.82±8.79	33.17±8.05	34.32±6.32	33.73±12.10	0.854
**Age 20–40 years (%)**	88 (80.0)	25 (83.3)	40 (85.1)	23 (69.7)	0.206
**Illiterate (%)**	29 (26.4)	12 (40.0)	8 (17.0)	9 (27.3)	0.082
**Rural (%)**	37 (33.6)	15 (50.0)	15 (31.9)	7 (21.2)	0.051
**Duration of combined-illness (months)**	10.87±16.96	7.60±9.51	11.61±19.53	12.80±18.28	0.446
**History of TB contact (%)**	30 (27.3)	4 (13.3)	12 (25.5)	14 (42.4)	0.029^*^
**Previous history of TB (%)**	46 (41.8)	11 (36.7)	20 (42.6)	15 (45.5)	0.772

* p<0.05 – significant

Clinical symptoms and signs are detailed in table 3. Cough was the most common symptom (91.8%) followed by LGF (85.5%), anorexia (82.7%), weight loss (66.4%), shortness of breath (55.5%), generalized weakness (53.6%), chest pain (28.2%), oral ulcers (26.4%) and others in study patients. Occurrence of various symptoms was nearly with similar frequency in three study groups with no statistically significant intergroup differences except for nausea +/− vomiting which was found with higher frequency in group I (36.7%) and group II (27.7%).

**Table 3.** Clinical features distribution in three groups of CD4 count

**Clinical Features**	**Total (n=110)**	**CD4≤100 (n=30)**	**CD4101–200 (n=47)**	**CD4>200 (n=33)**	**P value**
**Cough (%)**	101 (91.8)	28 (93.3)	42 (89.4)	31 (93.9)	0.716
**Low grade fever (%)**	94 (85.5)	23 (76.7)	42 (89.4)	29 (87.9)	0.273
**Anorexia (%)**	91 (82.7)	25 (83.3)	37 (78.7)	29 (87.9)	0.563
**Weight loss (%)**	73 (66.4)	19 (63.3)	37 (78.7)	27 (81.8)	0.185
**Shortness of breath (%)**	61 (55.5)	18 (60.0)	25 (53.2)	16 (48.5)	0.656
**Generalized weakness (%)**	59 (53.6)	14 (46.7)	23 (48.9)	22 (66.7)	0.196
**Chest Pain (%)**	31 (28.2)	7 (23.3)	15 (31.9)	9 (27.3)	0.710
**Oral Ulcers (%)**	29 (26.4)	7 (23.3)	13 (27.3)	9 (27.3)	0.906
**Nausea ± Vomiting (%)**	26 (23.6)	11 (36.7)	13 (27.7)	2 (6.1)	0.012^*^
**Loose Stool (%)**	25 (22.7)	8 (26.7)	7 (14.9)	10 (30.3)	0.225
**Lymph Node Swelling (%)**	23 (20.9)	7 (23.3)	8 (17.0)	8 (24.2)	0.685
**Dysphagia (%)**	19 (17.3)	4 (13.3)	8 (17.0)	7 (21.2)	0.710
**CNS Symptoms^#^ (%)**	15 (13.6)	7 (23.3)	4 (8.5)	4 (12.1)	0.173
**Constipation (%)**	13 (11.8)	4 (13.3)	7 (14.9)	2 (6.1)	0.462
**Temperature > 99°F (%)**	59 (53.6)	12 (40.0)	27 (57.4)	20 (60.6)	0.206
**SBP (%)**	112.27±17.7	107.20±17.16	112.68±15.06	116.30±20.85	0.123
**DBP (%)**	70.58±10.54	71.20±10.88	69.53±9.77	71.51±11.44	0.665
**BMI**	17.48±1.99	17.32±1.46	17.23±1.74	17.97±2.62	0.273
**BMI<18.0 (%)**	78 (70.9)	24 (80.0)	32 (68.1)	22 (66.7)	0.437

# CNS symptoms include altered sensorium, tremors, and seizures.

Lungs were the most common site of involvement (56.4%) followed next by combined pulmonary and extra-pulmonary involvement (28.2%). Extra-pulmonary only involvement was infrequent with only few patients noted in three groups. There was no statistically significant difference observed for involvement of any site amongst three groups (P=0.700) (Table 4). Frequency of sputum negative TB was higher in overall population as well as in three study groups. Though the difference in occurrence of sputum negative TB did not reach statistical significance among three groups (P=0.094), a considerably higher number of patients with CD4 counts below 100 had sputum negative TB (72%) as compared to sputum positive TB (28%).

**Table 4.** Site of involvement with TB and sputum AFB status in three groups

**Site of Involvement with TB**	**Total (n=110)**	**CD4 ≤100 (n=30)**	**CD4101–200 (n=47)**	**CD4>200 (n=33)**	**P value**
**Pulmonary**	62 (56.4)	15 (50.0)	28 (59.6)	19 (57.6)	0.700
**Pulmonary + Extra pulmonary**	31 (28.2)	10 (33.3)	14 (30.4)	7 (21.2)	
**Extra-pulmonary**	17 (15.5)	5 (16.7)	5 (10.9)	7 (21.2)	
**Extra-Pulmonary Sites**					
**Pleura**	20 (18.2)	7 (23.3)	7 (14.9)	6 (18.2)	-
**Lymph Node**	18 (16.4)	6 (20.0)	5 (10.6)	7 (21.2)	-
**Abdomen**	6 (5.5)	0	5 (10.6)	1 (3.0)	-
**CNS**	1 (0.9)	0	1 (2.1)	0	-
**Abdomen and Lymph node**	1 (0.9)	1 (3.3)	0	0	-
**Pericardium**	1 (0.9)	1 (3.3)	0	0	-
**Spine**	1 (0.9)	0	1 (2.1)	0	-
Sputum Status^*^	**N=93**	**N=25**	**N=42**	**N=26**	
**Sputum AFB Negative (%)**	50 (53.8)	18 (72.0)	19 (45.2)	13 (50.0)	0.094
**Sputum AFB Positive (%)**	43 (46.2)	7 (28.0)	23 (54.8)	13 (50.0)	

* Includes all cases with pulmonary involvement.

## DISCUSSION

In HIV positive patients, TB is the commonest opportunistic infection in India. Unlike other opportunistic infections which occur at CD4+ counts below 200/mm^3^, TB can crop up at any stage of the disease (23). The risk of developing clinical TB rises sharply after HIV infection, well before the total CD4 count drops to levels below 500 cells/mm^3^ (11). Although many studies have been done in western countries and African region to know the clinical profile of TB in HIV positive persons (24, 25); however, there is still a scarcity of such studies from India (26, 27).

Patients in present study had a mean and median CD4+ count of 181.7cells/cumm and 156, respectively. Median CD4 cell counts have ranged from < 150/cumm to > 300/cumm in previous reports of HIV-TB co-infected patients (28, 29).

Almost one third (30%) of our patients had CD4 counts <100 cells/mm^3^, whereas more than two third (70 %) of the patients had CD4 counts <200 cells/mm^3^, similar to other recent observations (30). In a study by Lee et al. most patients were in the advanced stage of HIV infection; 93% had CD4 cell count less than 200/mm^3^ (17). HIV is notoriously known to cause depletion and dysfunction of CD4 cells while TB may accelerate this process and a combination of both the infections could explain the reason, why the majority of patients in this study had low CD4 cell count. Another reason may be due to relatively delayed presentation of patients leading to severely immune-compromised state with some sort of opportunistic infection e.g. TB in our cases.

In our study, around 80% of study population belonged to sexually and economically productive age group of 21–40 years. Compared to females (29.1%), males (70.9%) were affected more frequently with significant difference observed amongst groups (P=0.013). Male predominance was also observed by Lee et al, who observed that majority of their study patients were male (88%) (17). Affected males belonged more commonly to groups II and III than group I, where females (46.9%) outnumbered the males, revealing the fact that females present more frequently with severe immune-suppression (Table 1). Mean age of the patients was 33.82±8.79 years, and it was not significantly different in three groups based on CD4 count. Similarly, a meta-analysis of European studies found that the majority of their co-infected cases were male and young adults (aged 30 to 45 years) (30). Previous studies show that patients co-infected with HIV-TB presents late to the health care facility. The delay between the onset of symptoms and the final diagnosis of co-infection may range from 12 weeks to more than 6 months (4, 24). Our patients had an average total duration of illness of 10.87±16.96 months, with marked deterioration in last 1–2 months but the duration of combined illness did not vary significantly among the groups. Clearly there was a delay in diagnosing TB and starting treatment and it was probably because of very poor education, lower awareness for diseases, and poorly established healthcare and referral system in this tribal area of the state. To know exactly, whether the delay was at the patient or provider level, needs further investigation.

In our study, the classical symptom of pulmonary TB, cough either dry or productive topped the list of respiratory symptoms (91.8%), followed by non-localizing symptoms like low grade fever (85.5 %), loss of appetite (82.7 %), and weight (66.4 %). Frequency of various symptoms was nearly similar in three study groups with no statistically significant intergroup differences. Similar findings were also noted by other authors in the past (4, 17). Contrary to this, some authors have reported that fever and weight loss are more common, while cough and hemoptysis are less common in HIV-PTB co-infected patients than HIV-negative probably because of less cavitation, inflammation and endobronchial irritation in HIV-positive patients (31). More than half of the patients (53.6%) were febrile on examination. Overall 70.9% patients were in undernourished category with higher number of patients in group I (80%) being undernourished than in other two groups while the mean BMI in all three groups was in malnourished category. Thus, the presenting symptoms of TB in HIV infected persons are not different from those in non-HIV infected persons. Recently Kim et al. have also recommended that these findings may also be used for identifying people at risk of having TB in HIV care settings (32).

Clinical presentation of TB in HIV-infected patients primarily relies on the level of immunosuppression caused by HIV infection (28). Pulmonary TB (PTB) is more frequently seen than extrapulmonary TB (EPTB) in patients with relatively intact immune function (CD4+ count > 200/mm^3^) (28, 33). As immune deficiency advances, HIV-infected patients present with atypical pulmonary disease resembling primary TB or because of weakened immune system which is less capable to stop the growth and spread of tubercle bacilli, leads to increased chances of extrapulmonary and disseminated disease (24, 34). Since the HIV epidemic commenced much earlier in this area of Rajasthan due to proximity with high prevalence states of Gujarat and Maharashtra, more than half of our HIV patients were in the late phase of disease and a wide clinical spectrum of TB was observed among HIV patients depending on their varying immune status.

Even in HIV-infected patients, pulmonary TB is still the commonest form of TB. We found pulmonary involvement by *M. tuberculosis* in 84.6% of HIV infected individuals; of whom 56.4% had pulmonary TB component alone and 28.2 % patients had simultaneously involved any of the extra-pulmonary sites. Only 15.5% patients had exclusively extra-pulmonary TB while extra-pulmonary involvement was seen in a total of 43.7% patients. There was no statistically significant difference observed for involvement of any site amongst three groups (P=0.700) (Table 4). A study from the same region previously reported isolated pulmonary disease in 45%, extra-pulmonary disease in 25% and mixed in 30%, while the rates of extra-pulmonary involvement were higher than these in other studies ranging from 31% to 75.8% (27). The explanation for this fact is probably HIV-positive patients who live in TB endemic areas, as is the case of the area where the present study was conducted, acquire TB either before HIV infection or earlier in the course of disease, when their immunity is still preserved, and more likely to develop typical forms of the disease e.g. pulmonary TB (26).

Study from Hong-Kong observed 37% patients with pulmonary involvement alone, 13% had extra-pulmonary disease, and 50% had both pulmonary and extrapulmonary involvement (17). Similarly, a study from USA also found only 38% of TB in the lungs alone, while 30% entirely extra-pulmonary and 32% both pulmonary and extrapulmonary (35). As immunosuppression worsens, HIV-infected patients may more often present with atypical features such as lymphadenopathy, pleural effusion, pericardial effusion, miliary TB, meningitis, and disseminated TB (24, 34).

Studies have shown that tuberculous lymphadenopathy, both intra- and extra thoracic, remain the most frequent form of extrapulmonary TB (17). Similarly, we also found lymph node TB (16.4%) and pleural effusion (18.2%) followed by abdominal TB (5.5%). Similarly Frieden et al. observed extra-pulmonary TB in lymph nodes (43%), pleura (23%), central nervous system (8%), and genitourinary tract (5%) patients (10). TB is often disseminated involving two or more non-contiguous organs/sites concomitantly, in patients with HIV (36). In our study 5.5% patients had disseminated TB in contrast to 25% patients in a study from Hong-Kong, probably due to the patient of that study had a significantly lower mean CD4 cell count (mean 40) (17).

TB, both pulmonary and extra-pulmonary were more prevalent in HIV patient who presented with lower CD4 cell counts (<200 cells/mm^−3^) at our institute, similar to previous observations (37). Laboratory diagnosis of TB in HIV infected patients has been very challenging especially in poor resource settings because of scarcity of newer diagnostic methods. Because sputum smear remains the cornerstone of rapid diagnosis of TB in much of the world, even in areas of high HIV prevalence, including India. But the diagnostic accuracy of sputum smear microscopy is substantially impaired in patients with TB-HIV co-infection making diagnosis difficult (21–22), and its performance is even worst in patients with advanced immune suppression (18).

Also due to operator dependence on sample collection and processing, and interpretation of sputum smears, the sensitivity of the sputum smear may vary greatly in resource-constrained settings (4). In our study population also, frequency of sputum negative TB was higher in overall population as well as in three study groups and sputum was positive for AFB smear in only 46.2% patients of pulmonary involvement. Thus, though the difference in occurrence of sputum negative TB did not reach statistical significance among three groups (p=0.094), a considerably higher number of patients with CD4 counts below 100 had sputum negative TB (72%) as compared to sputum positive TB (28%) (Table 4). These smear-negative patients often are not diagnosed early, leading to higher mortality in such cases. Similar findings were observed by other authors who found sputum positivity ranging from 36% to 61% (17). Prevalence of sputum smear positivity in HIV-TB patients primarily depends on the degree of immune-suppression*.* Likelihood of positive sputum smear in mildly immunocompromised is similar to HIV-negative patient while in severely immunocompromised, it is decreased (due to decreased inflammation in lungs) (18). Although our study population had a similar prevalence (53.8%) of AFB smear negative disease compared to past estimates of 30–50% (28, 38), likely because of the fact that our institute's role as a referral centre and patients present late in course of disease, in which the prevalence of AFB smear-negative PTB may exceed 50%. Further, smear-positive HIV-TB patients tend to excrete very few organisms in sputum than HIV-negative patients, which can lead to acid-fast bacilli being missed, and falsely diagnosed as smear negative disease (24).

Most of extra-pulmonary TB whether it is abdominal (83.4%), pleural (70%), lymphadenopathy (61.1%), CNS or disseminated TB had CD4 counts below 200, in consonance with the previous studies which show that atypical presentation of TB increase with reduction in CD4 counts (18). Further, patients with a combination of pulmonary and extra-pulmonary TB had significantly lower CD4 counts and 77.4% of patients had CD 4 counts below 200 (39). As clinically detected active TB is associated with worse clinical outcomes, greater mortality and morbidity, identifying HIV-TB co-infected patients, earlier in the HIV-disease trajectory may halt TB-related morbidity and mortality (40).

The strengths of our study include analysis of clinical TB and also assessing these presentations across a resolution of CD4 strata than has not been previously published. A unique feature of our study was the assessment of clinical presentation of TB, whether pulmonary, extrapulmonary or combined, varying with the level of immune-suppression and correlation of these findings with CD4 levels, a feature previously not described.

Our study has some limitations also, including bias in patient selection. As our centre is a referral centre for the region, it is highly likely that patients with TB seen here, have more severe or clinically advanced disease due to delayed referral from peripheral areas. Selective referral for positive patients may explain the relatively low prevalence of AFB smear-negative PTB. As the symptom screening and sputum microscopy perform poorly in this group of patients, most requiring rapid TB diagnosis, chances are higher for misdiagnosis, this study may help somewhat in improvement in our understanding of the impact of gradual immunosuppression on the presentation of TB, which allows peripherally based clinicians to suspect their patients' likelihood of having TB earlier and thus reducing the diagnostic limitations in HIV-TB co-infected patients.
